# Supplementary material for: Content-rich biological network constructed by mining PubMed abstracts
Source: BMC Bioinformatics. 2004 Oct 8;5:147. doi: 10.1186/1471-2105-5-147 (PMC528731; doi:10.1186/1471-2105-5-147)
Supplement: Additional File 2 — The original results of the above study (non-essential files are deleted to keep the file size under the limit set by BMC bioinformatics). [file 1471-2105-5-147-S2.bz2 › chilibotAdditionalFile2/dip50/391ID10498602E9873/html/NRP1_SEMA3A.html]

 


 **NRP1** and **SEMA3A** 
  
Found 69 abstracts in PubMed, retrieved 50.  
 

 What does Google say? 
 PDF only 
| .edu only 

---

**Interactive relationship** (e.g. stimulation, inhibition, etc)

**Inhibitory relationship**- VEGF165 and  **Sema3A**  are competitive inhibitors for  **NRP1**  mediated functions in EC and neurons.  Ref: 12613541 Adv Exp Med Biol, 2002
- We show that the misexpression of a dominant negative neuropilin 1  [ **NRP1** ]  that blocks SEMA  [ **SEMA3A** ]  3A mediated signaling in olfactory sensory axons induces many of them to enter the tel encephalon prematurely and to overshoot the olfactory bulb.  Ref: 11144354 Neuron, 2000
- In the presence of  **Sema3A** , these oligodendrocytes displayed a dramatic reduction in process extension, which was reversed by removal of  **Sema3A**  and prevented by anti neuropilin 1  [ **NRP1** ] , anti Ulip6 CRMP5, anti Ulip2 CRMP2 antibodies, or VEGF 165, another neuropilin 1  [ **NRP1** ]  ligand.  Ref: 11549731 J Neurosci, 2001
- However, the response of growth cones belonging to these neurons induced by recombinant collapsin 1  [ **SEMA3A** ]  semaphorin3a and blocked by the anti neuropilin 1  [ **NRP1** ]  antibody was restricted to those of small cell body diameter < ; 30 microm, corresponding primarily to nociceptive sensory afferents.  Ref: 10588387 Mol Cell Neurosci< MedlineTA>Mol Cell Neurosci, 2001
**Neutral relationship**- Two neuropilin 1  [ **NRP1** ]  ligands, semaphorin III  [ **SEMA3A** ]  and VEGF 165, can bind to these cells, and the addition of VEGF 165 to MS 5 cells increases expression of 2 cytokines known to regulate early hematopoiesis, Tpo and Flt3 L.  Ref: 10498602 Blood, 1999
- Semaphorin 3A  [ **SEMA3A** ]   **Sema3A**  binds to neuropilin 1  [ **NRP1** ]  NP1 and activates the transmembrane Plexin to transduce a repulsive axon guidance signal.  Ref: 11239433 Neuron, 2001
- Neuropilin 1  [ **NRP1** ]  is the specific receptor for  **Sema3A**  and plays a role in nerve fiber guidance.  Ref: 11830568 Development, 2002
- Plexin neuropilin 1  [ **NRP1** ]  complexes form functional semaphorin 3A  [ **SEMA3A** ]  receptors.  Ref: 10520994 Cell, 1999
- **Sema3A**  binds with high affinity to neuropilin 1  [ **NRP1** ]  on growth cone filopodial tips.  Ref: 10934324 J Neurobiol, 2000
- Cis and trans interactions of L1 with neuropilin 1  [ **NRP1** ]  control axonal responses to semaphorin 3A  [ **SEMA3A** ] .  Ref: 12456642 EMBO J, 2002
- COS 7 cells coexpressing plexin A4 with neuropilin 1  [ **NRP1** ]  were induced to contract by  **Sema3A** , a member of the class 3 semaphorin.  Ref: 12591607 Mech Dev, 2003
- The fetal lung explants from neuropilin 1  [ **NRP1** ]  homozygous null mice grew normally in vitro regardless of  **Sema3A**  treatment.  Ref: 11025205 Mech Dev, 2000
- Characterization of neuropilin 1  [ **NRP1** ]  structural features that confer binding to semaphorin 3A  [ **SEMA3A** ]  and vascular endothelial growth factor 165.  Ref: 11886873 J Biol Chem, 2002
- From E7 until E10 NT3 responsive growth cones expressed progressively lower levels of neuropilin 1  [ **NRP1** ] , and were less sensitive to  **Sema3A** .  Ref: 11920727 J Neurobiol, 2002
- Neuropilin 1  [ **NRP1** ]  Npn 1 is a receptor for both semaphorin 3A  [ **SEMA3A** ]   **Sema3A**  and vascular endothelial growth factor 165 VEGF 165.  Ref: 11886873 J Biol Chem, 2002
- During development, neuropilin 1  [ **NRP1** ]  is a receptor for semaphorin 3a  [ **SEMA3A** ]  mediated axonal guidance and for vascular endothelial growth factor VEGF promotion of angiogenesis.  Ref: 11333364 J Cereb Blood Flow Metab, 2001
- Semaphorin 3A  [ **SEMA3A** ]   **Sema3A**  repels growing olfactory axons that express neuropilin 1  [ **NRP1** ]  NP 1, a receptor for  **Sema3A** .  Ref: 12598627 J Neurosci, 2003
- Expression of sema3a1 in somites and neuropilin 1  [ **NRP1** ] , which encodes for a component of the  **Sema3a**  receptor, in angioblasts suggested that Sema3a1 regulates the pathway of the dorsally migrating angioblasts.  Ref: 12783793 Development< MedlineTA>Development, 2003
- The effects of one of these repulsive cues, semaphorin 3A  [ **SEMA3A** ]   **Sema3A** , are mediated by the membrane protein neuropilin 1  [ **NRP1** ]  Npn 1.  Ref: 10781943 Mech Dev, 2000
- Neuropilin 1  [ **NRP1** ]  NP 1 is a component of the receptor for semaphorin3a  **Sema3a** , a member of a large family of molecules with widespread expression and demonstrable influence via their ability to repel growing axons on nervous system development.  Ref: 10870088 J Comp Neurol, 2000
- These results indicate the existence in the adult brain of a  **Sema3A**  signaling pathway that modulates oligodendrocyte process extension mediated by neuropilin 1  [ **NRP1** ] , Ulip6 CRMP5, and Ulip2 CRMP2, and they open new fields of investigation of neuron oligodendrocyte interactions in the normal and pathological brain.  Ref: 11549731 J Neurosci, 2001
- Furthermore, the  **Sema3A**  receptor proteins, neuropilin 1  [ **NRP1** ]  and plexin, and the  **Sema3A**  signaling molecule, rac1, also reorganize to vacuoles and membrane ridges after  **Sema3A**  treatment.  Ref: 10769032 J Cell Biol, 2000
- The axon guidance signal semaphorin 3A  [ **SEMA3A** ]  induces the rapid collapse of growth cones by activating a receptor complex that contains neuropilin 1  [ **NRP1** ]  as the ligand binding and a plexin as the signal transducing subunit.  Ref: 11108845 FEBS Lett, 2000
- Neuropilin 1  [ **NRP1** ]  Npn 1 is a receptor that binds multiple ligands from structurally distinct families, including secreted semaphorins Sema  [ **SEMA3A** ]  and vascular endothelial growth factors VEGF.  Ref: 12852851 Dev Cell, 2003

**Non-interactive relationship** (e.g. studied together, co-existance, homology, etc.)

- To assess the role of semaphorin 3A  [ **SEMA3A** ]   **Sema3A**  and its receptor component neuropilin 1  [ **NRP1** ]  Npn 1 in pontocerebellar axon guidance, we compared the distributions of  **Sema3A** , Npn 1, and DiI labeled pontocerebellar axons in neonatal mouse cerebellum.  Ref: 12401447 Mol Cell Neurosci, 2002
- Neuropilin 1  [ **NRP1** ] , a neuronal cell surface semaphorin III  [ **SEMA3A** ]  receptor protein important for axonal guidance in developing peripheral nervous system efferents, has also been identified as a vascular endothelial growth factor VEGF receptor on endothelial cells.  Ref: 10919846 Am J Physiol Renal Physiol, 2000
- Neuropilin 1  [ **NRP1** ]  is expressed on adult mammalian dorsal root ganglion neurons and mediates semaphorin3a collapsin 1  [ **SEMA3A** ]  induced growth cone collapse by small diameter sensory afferents.  Ref: 10588387 Mol Cell Neurosci< MedlineTA>Mol Cell Neurosci, 2000
- Semaphorin 3A  [ **SEMA3A** ]   **Sema3A**  is a membrane associated secreted protein that has chemorepulsive properties for neuropilin 1  [ **NRP1** ]  npn 1 expressing axons.  Ref: 11027230 J Neurosci, 2000
- **Sema3A** , a ligand for neuropilin 1  [ **NRP1** ] , is effective in repelling geniculate and trigeminal axons, and antineuropilin 1, but not antineuropilin 2, completely blocks the repulsion by arch explants that repel axon outgrowth from both ganglia.  Ref: 10861527 J Comp Neurol, 2000
- We report that neuropilin 1  [ **NRP1** ]  and  **Sema3A**  mutant mouse embryos, generated by targeted gene disruption, showed displacement of sympathetic neurons and their precursors and abnormal morphogenesis in the sympathetic trunk.  Ref: 11830568 Development, 2002
- Expression of mRNAs of  **Sema3A**  and neuropilin 1  [ **NRP1** ]  NP 1, a  **Sema3A**  receptor, was detected in fetal and adult lungs.  Ref: 11025205 Mech Dev, 2000
- The dermamyotome derived chemorepulsion is exclusively dependent on  **Sema3A**  neuropilin 1  [ **NRP1** ] .  Ref: 12591248 Dev Biol, 2003
- Second, L1 and  **NRP1** , the neuropilin responsible for  **Sema3A**  binding, associate through their extracellular domains, forming a cell surface heterocomplex.  Ref: 12613546 Adv Exp Med Biol, 2002
- We also show that  **Sema3A**  suppressed the cell migration activity of sympathetic neurons from wild type but not neuropilin 1  [ **NRP1** ]  mutant embryos in vitro and instead promoted their accumulation into compact cell masses and fasciculation of their neurites.  Ref: 11830568 Development, 2002
- Growth cones extended from E6 DRGs in NT3 containing medium expressed neuropilin 1  [ **NRP1** ]  and collapsed in response to  **Sema3A** .  Ref: 11920727 J Neurobiol, 2002
- Both repulsion and induction of cell death are mediated by neuropilin 1  [ **NRP1** ] , the ligand binding component of the  **Sema3A**  receptor.  Ref: 11331362 J Neurosci, 2001
- We compared mRNA expression of class 3 semaphorins  **Sema3A** , 3B, 3C and 3F and their two receptors Neuropilin 1  [ **NRP1** ]  and 2 in the embryonic mouse first molar tooth germ E10 18 by radioactive in situ hybridization.  Ref: 11231073 Mech Dev, 2001
- BACKGROUND Previously, we reported that  **Sema3A** , one of the secreted repulsive axon guidance molecules, CRMP collapsin response mediator protein 2, a putative intracellular signalling molecule for  **Sema3A**  and  **Sema3A**  receptor neuropilin 1  [ **NRP1** ]  are expressed in the developing lung.  Ref: 11442635 Genes Cells, 2001
- This review focuses on semaphorin 3A  [ **SEMA3A** ]  probably the best characterized semaphorin and its receptors in particular neuropilin 1  [ **NRP1** ]  in the adult nervous system and argues that semaphorin 3A  [ **SEMA3A** ]  plays a role in the maintenance and regeneration of adult sensory neurons.  Ref: 11545265 Cell Tissue Res, 2001
- This study compared the expression of the semaphorin 3A  [ **SEMA3A** ]  receptor subunit, neuropilin 1  [ **NRP1** ] , and the collapse response of growth cones to semaphorin 3A  [ **SEMA3A** ]  for NGF cutaneous and NT3 proprioceptive dependent sensory axons extended from E6 E10 chick embryos.  Ref: 11920727 J Neurobiol, 2002
- Instead, the  **Sema3A**  neuropilin 1  [ **NRP1** ]  complex interacts with another transmembrane protein, plexin, on the surface of growth cones.  Ref: 10934324 J Neurobiol, 2000
- Requirement of neuropilin 1  [ **NRP1** ]  mediated  **Sema3A**  signals in patterning of the sympathetic nervous system.  Ref: 11830568 Development, 2002
- Thus, developmental patterning of sensory terminals in the chick spinal cord may arise from changes in both  **Sema3A**  expression in the developing spinal cord and accompanying changes in neuronal expression of the  **Sema3A**  receptor subunit, neuropilin 1  [ **NRP1** ] .  Ref: 11920727 J Neurobiol, 2002
- Expressions of neuropilin 1  [ **NRP1** ] , neuropilin 2 and semaphorin 3A  [ **SEMA3A** ]  mRNA in the rat brain after middle cerebral artery occlusion.  Ref: 11578592 Brain Res, 2001
- Using coculture assays composed of tissues derived from the chick embryo or mutant mice treated with function blocking antibodies and phosphatidylinositol specific phospholipase C, we report here that the chemorepellent semaphorin 3A  [ **SEMA3A** ]   **Sema3A**  and its receptor neuropilin 1  [ **NRP1** ]  are required for mediating the dermamyotome and notochord derived, but not the ventral spinal cord derived, chemorepulsive signal for DRG axons.  Ref: 12591248 Dev Biol, 2003
- Neuropilin 1  [ **NRP1** ]  and plexin As form receptor complexes for  **Sema3A**  and plexin As are thought to initiate the intracellular signaling cascade.  Ref: 12559962 Biochem Biophys Res Commun, 2003
- These findings suggest that the neuropilin 1  [ **NRP1** ]  mediated  **Sema3A**  signals regulate arrest and aggregation of sympathetic neuron precursors and sympathetic neurons themselves at defined target sites and axon fasciculation to produce the stereotyped sympathetic nerve pattern.  Ref: 11830568 Development, 2002
- Oligodendrocytes were found to have  **Sema3A**  binding sites and to express neuropilin 1  [ **NRP1** ] , the major  **Sema3A**  receptor component.  Ref: 11549731 J Neurosci, 2001
- Among its binding partners, L1 immunoglobulin superfamily adhesion molecule Ig CAM associates with neuropilin 1  [ **NRP1** ]  NP 1 to form a semaphorin3A  **Sema3A**  receptor and soluble L1 converts  **Sema3A**  induced axonal repulsion into attraction.  Ref: 12456642 EMBO J, 2002
- After the initial discovery of neuropilin 1  [ **NRP1** ]  as an epitope on axons recognized by a monoclonal antibody, neuropilins were rediscovered in the search for receptors mediating the repulsive actions of class 3 Semaphorins, notably  **Sema3A** .  Ref: 12613540 Adv Exp Med Biol, 2002
- Recent work has shown that neuropilin 1  [ **NRP1** ]  is essential but not sufficient to form functional  **Sema3A**  receptors and indicates that additional components are required to transduce signals from the cell surface to the cytoskeleton.  Ref: 10781943 Mech Dev, 2000
- These defects in Brn3a mice resemble strongly those seen in mice lacking the mediators of sensory pathfinding semaphorin 3A  [ **SEMA3A** ]  and neuropilin 1  [ **NRP1** ] .  Ref: 11160433 J Neurosci, 2001
- Temporal regulation of neuropilin 1  [ **NRP1** ]  expression and sensitivity to semaphorin 3A  [ **SEMA3A** ]  in NGF and NT3 responsive chick sensory neurons.  Ref: 11920727 J Neurobiol, 2002
- This neurotrophin mediated decrease in the collapse response of DRG growth cones was not associated with reduced expression on growth cones of the  **Sema3A**  binding protein neuropilin 1  [ **NRP1** ] .  Ref: 12151545 J Neurosci, 2002
- On the other hand, growth cones extended from DRGs in NGF containing medium expressed progressively higher levels of neuropilin 1  [ **NRP1** ]  and higher levels of collapse response to  **Sema3A**  over the period from E6 E10.  Ref: 11920727 J Neurobiol, 2002
- Consistent with the insensitivity of trkC collaterals to  **SEMA3A** , these collaterals did not express neuropilin 1  [ **NRP1** ] , a receptor for  **SEMA3A** .  Ref: 11077427 J Neurobiol, 2000
- Although neuropilin 1  [ **NRP1** ]  is required for  **Sema3A**  action, it is incapable of transmitting a  **Sema3A**  signal to the growth cone interior.  Ref: 10934324 J Neurobiol, 2000
- Chemorepulsion from the ventral spinal cord does not depend on  **Sema3A**  neuropilin 1  [ **NRP1** ]  but requires axonin 1 SC2 to repel DRG axons.  Ref: 12591248 Dev Biol, 2003
- Pontocerebellar axon guidance neuropilin 1  [ **NRP1** ]  and semaphorin 3A  [ **SEMA3A** ]  sensitivity gradients across basilar pontine nuclei and semaphorin 3A  [ **SEMA3A** ]  variation across cerebellum.  Ref: 12401447 Mol Cell Neurosci, 2002
